# Supplementary material for: Tuning the Morphology in the Nanoscale of NH4CN Polymers Synthesized by Microwave Radiation: A Comparative Study
Source: Polymers (Basel). 2021 Dec 24;14(1):57. doi: 10.3390/polym14010057 (PMC8747084; doi:10.3390/polym14010057)
Supplement: Supplementary file 1 [file polymers-14-00057-s001.zip › polymers-1469707-supplementary.pdf]

TUNING THE MORPHOLOGY IN THE NANOSCALE OF NH_4CN POLYMERS SYNTHESIZED BY MICROWAVE RADIATION: A COMPARATIVE STUDY

Cristina Pérez-Fernández, Pilar Valles, Elena González-Toril, Eva Mateo-Martí, José L. de la Fuente, Marta Ruiz-Bermejo

SUPPLEMENTARY INFORMATION

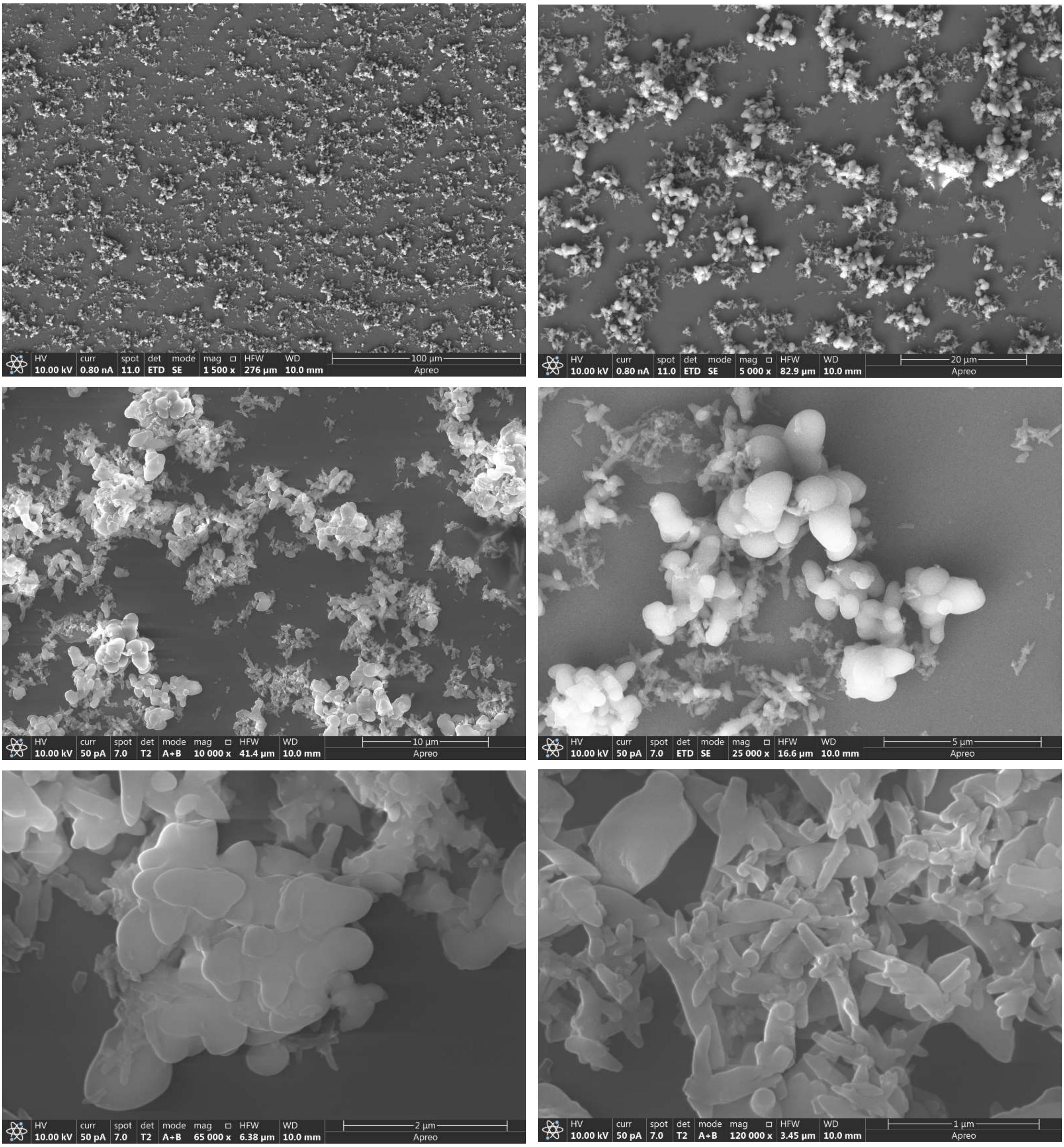

Figure S1. SEM images of the NH_4CN polymer **1**.

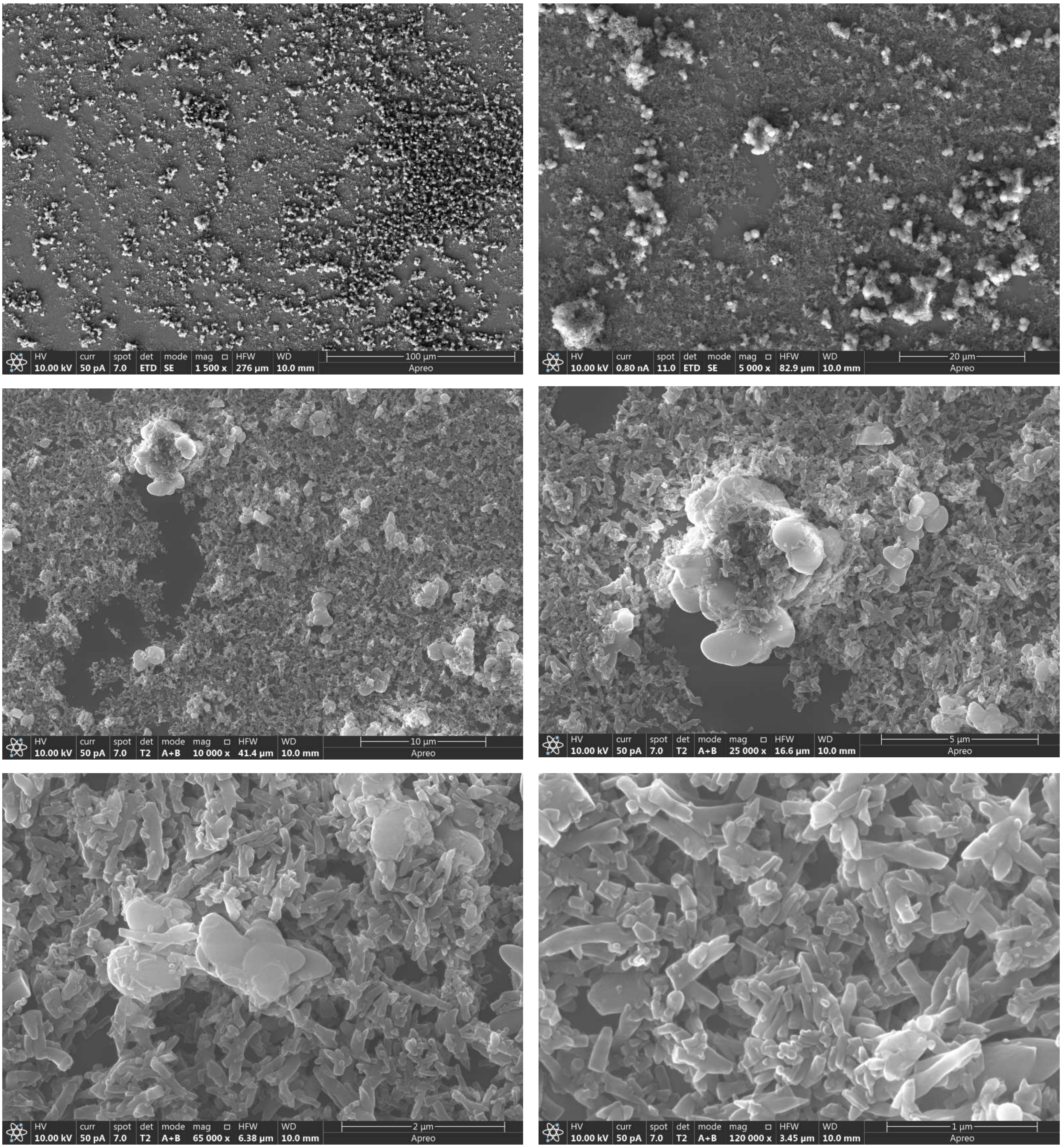

Figure S2. SEM images of the NH_4CN polymer **2**.

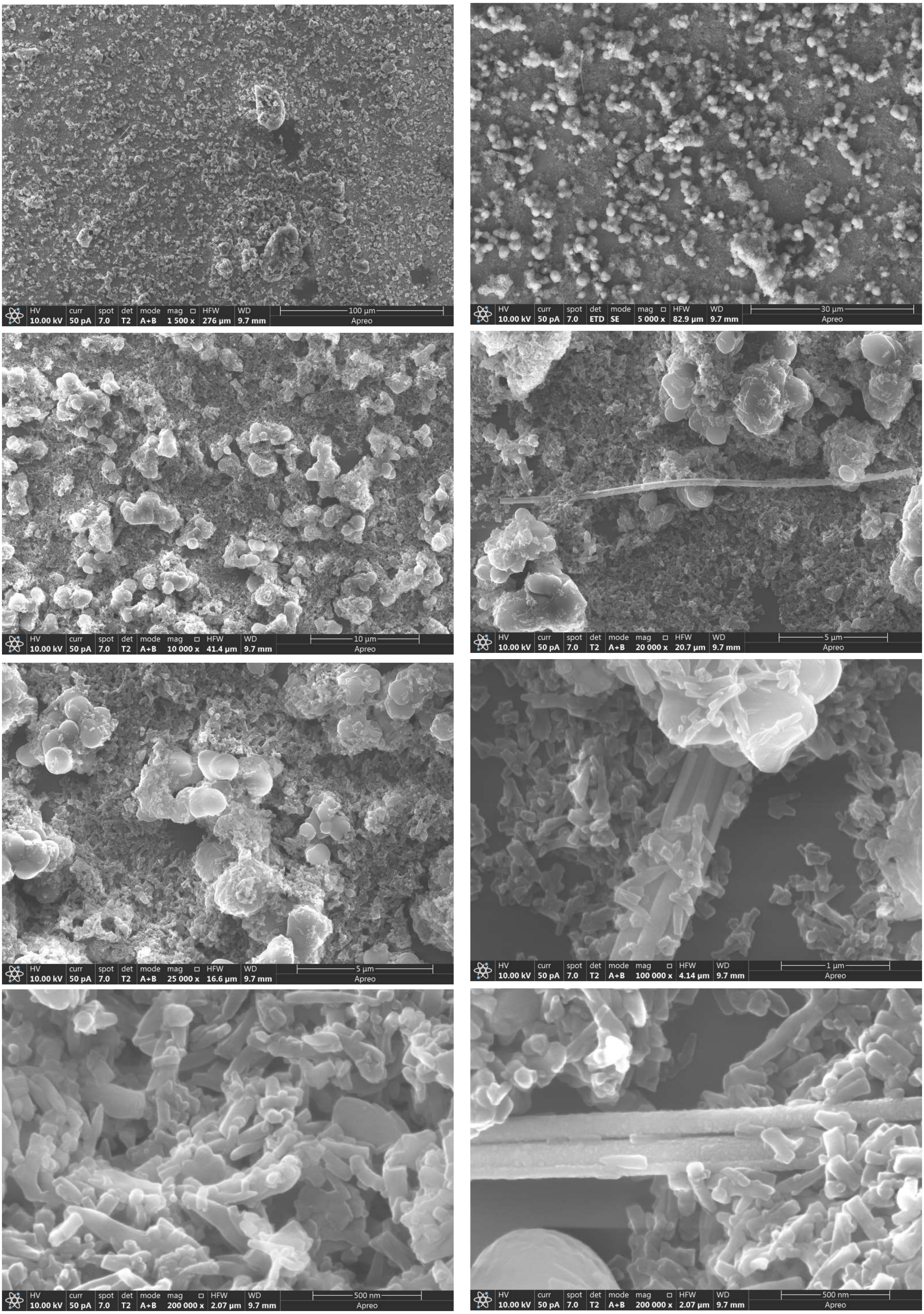

Figure S3. SEM images of the NH_4CN polymer **3**.

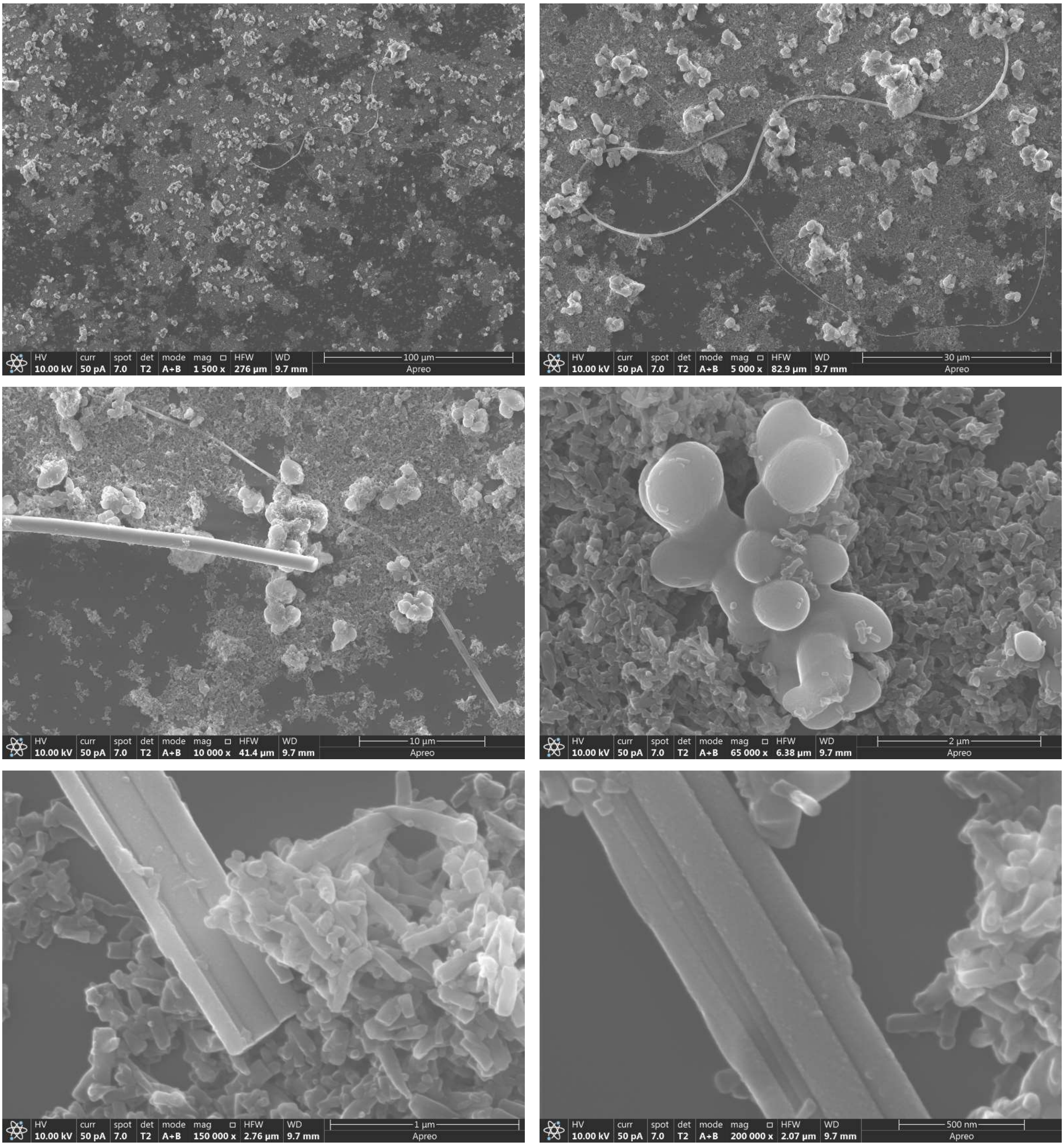

Figure S4. SEM images of the NH_4CN polymer **4**.

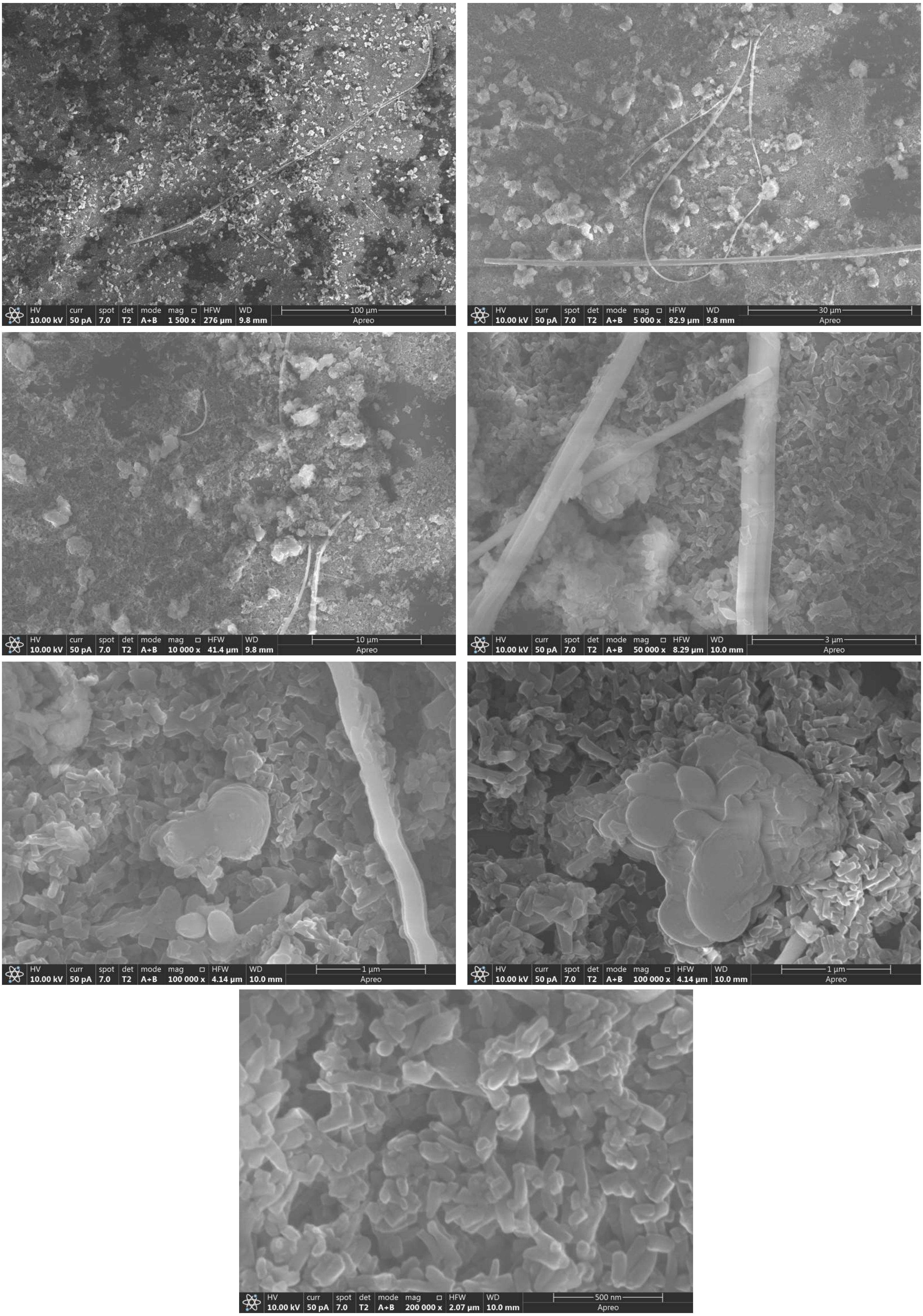

Figure S5. SEM images of the NH_4CN polymer **5**.

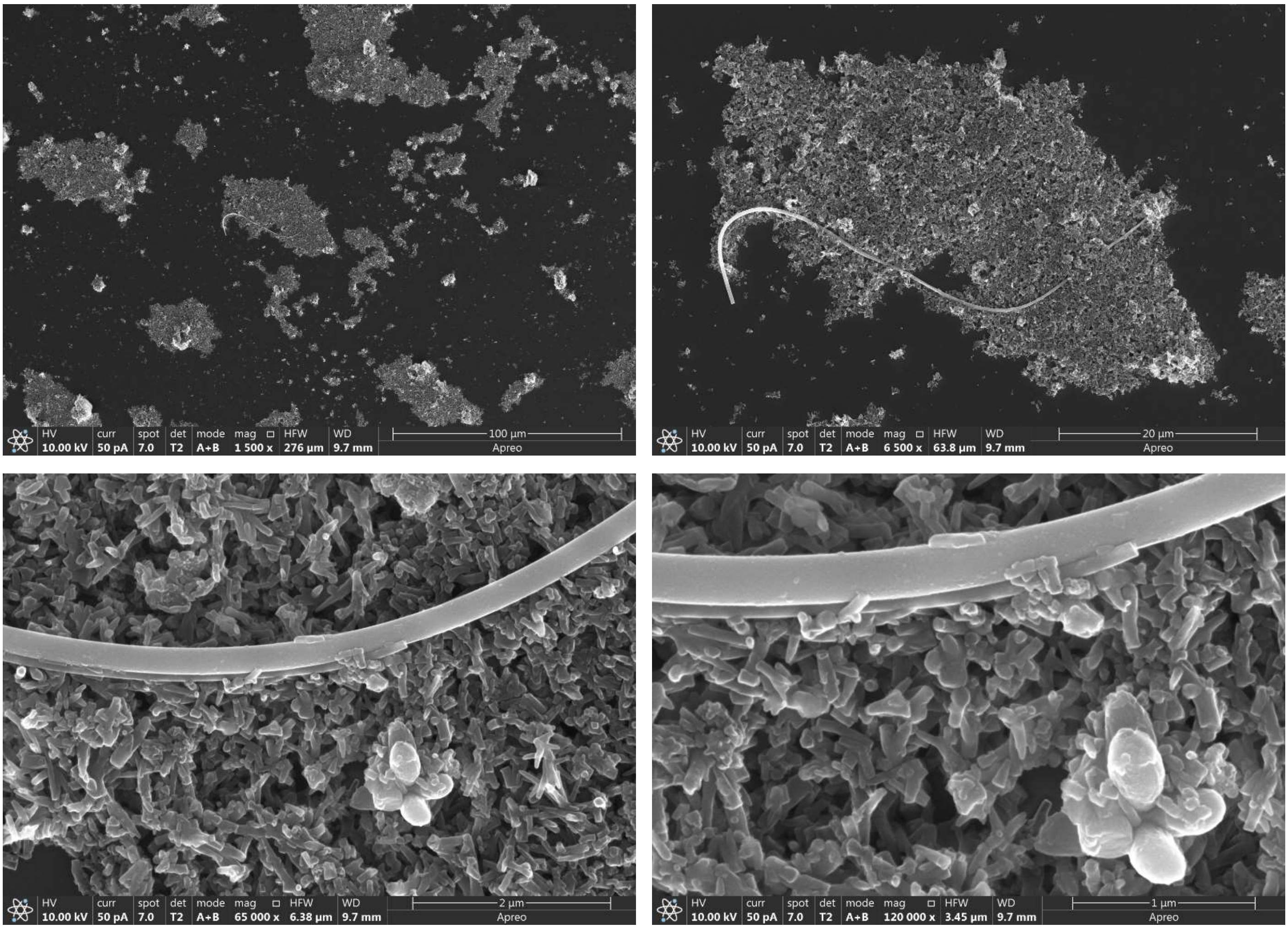

Figure S6. SEM images of the NH_4CN polymer **6**.

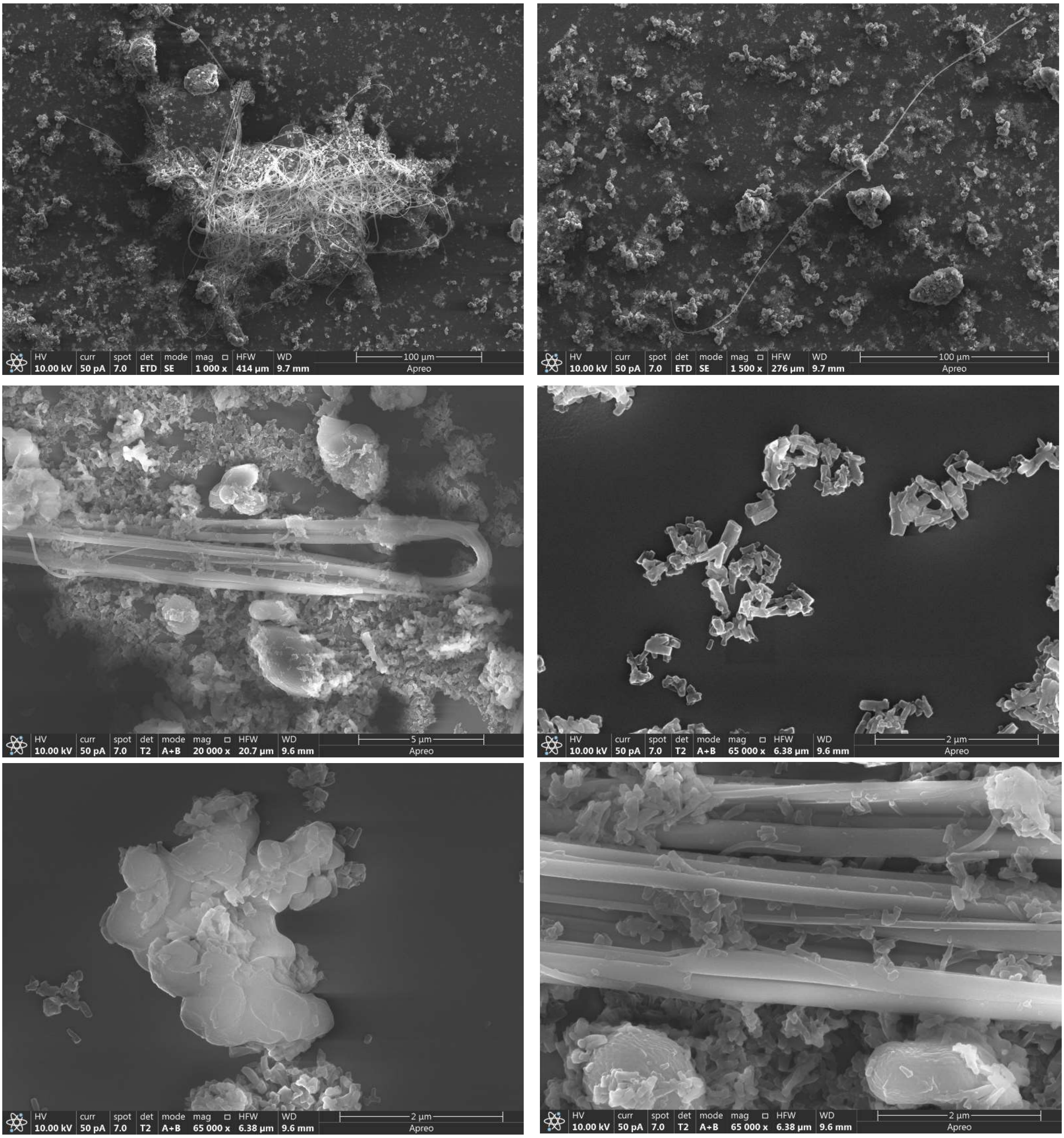

Figure S7. SEM images of the NH_4CN polymer **7**.

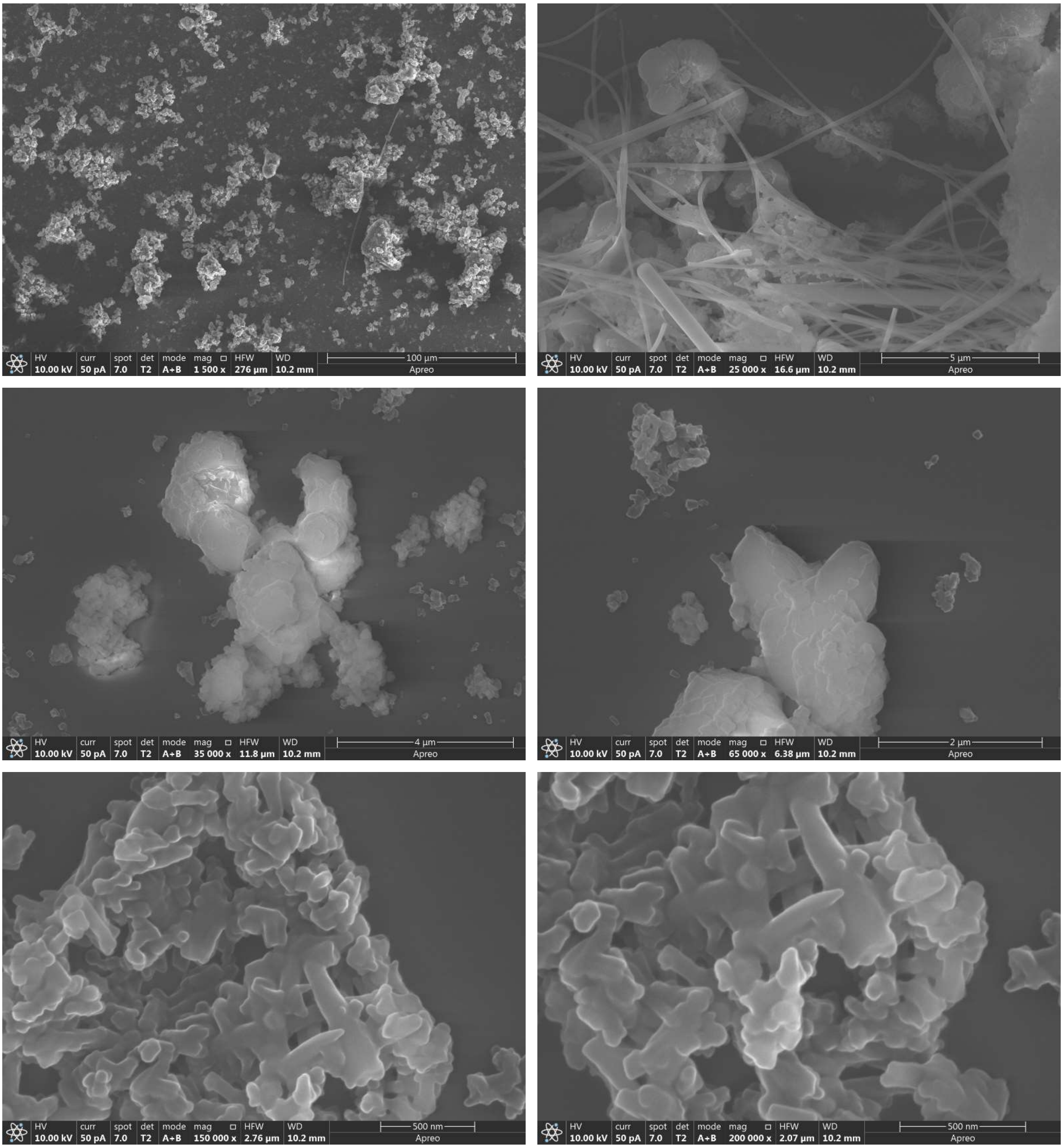

Figure S8. SEM images of the NH_4CN polymer **8**.

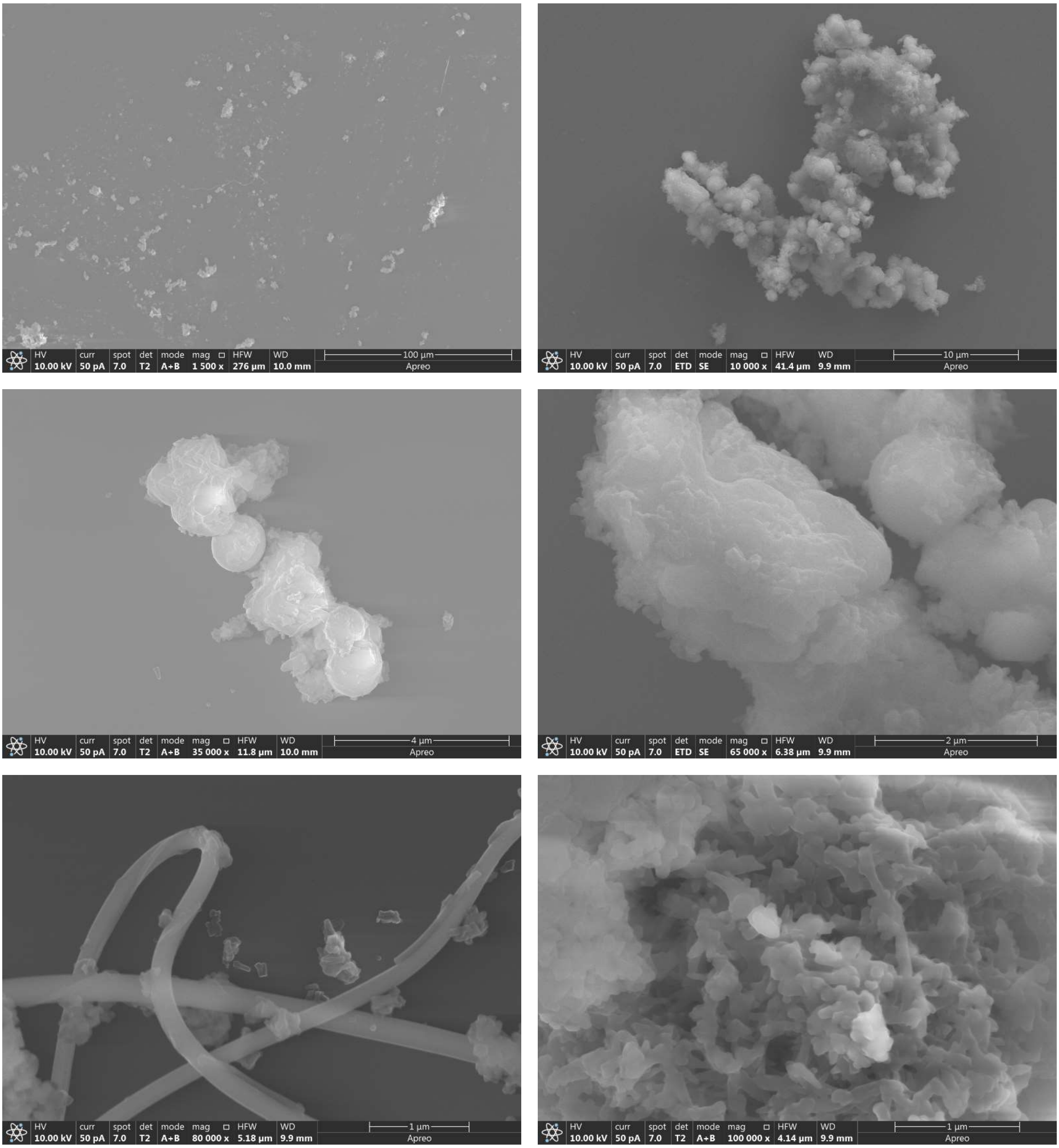

Figure S9. SEM images of the NH_4CN polymer **9**.

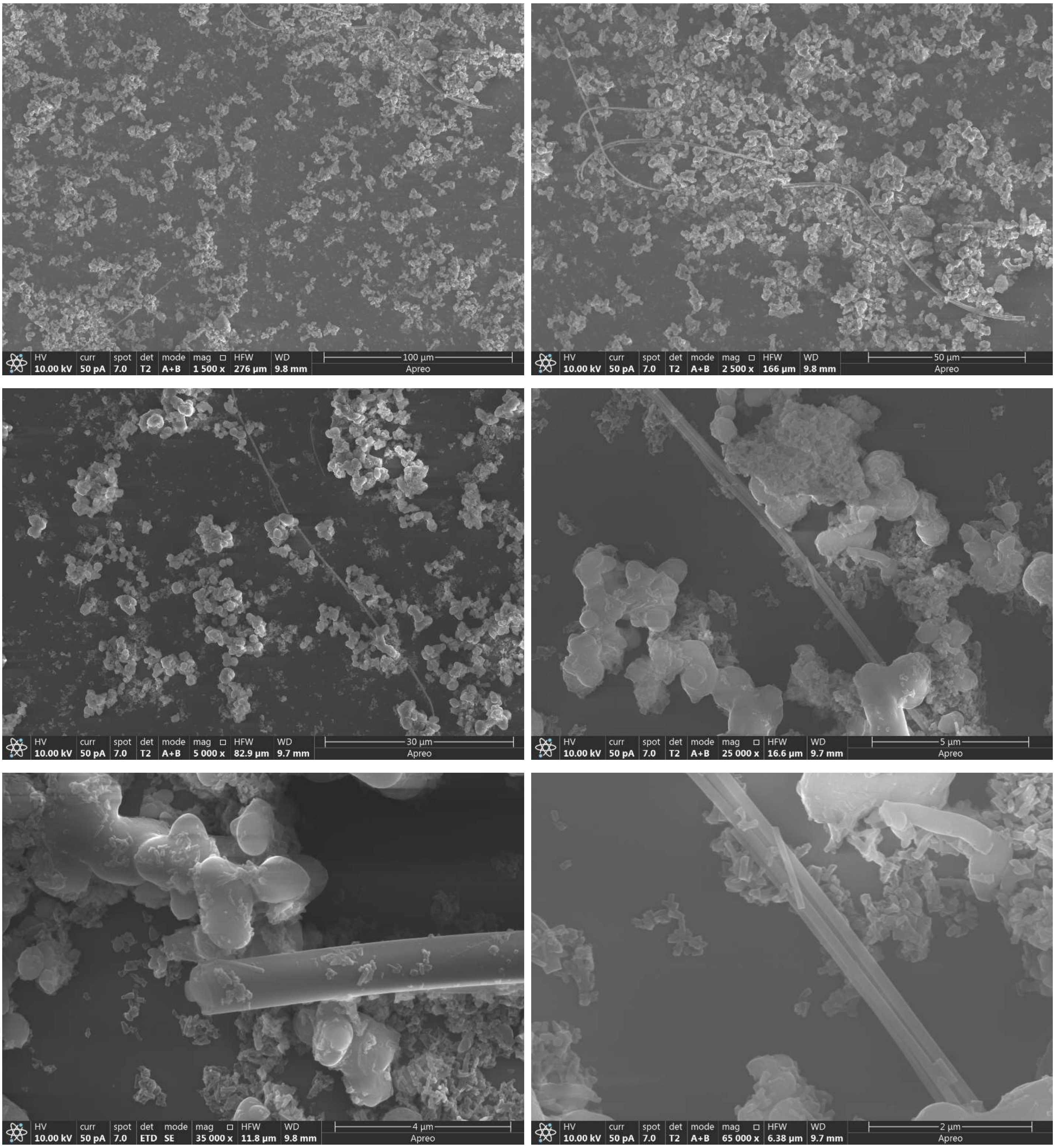

Figure S10. SEM images of the NH_4CN polymer synthetized at 170 ºC with a reaction time of 5 min.

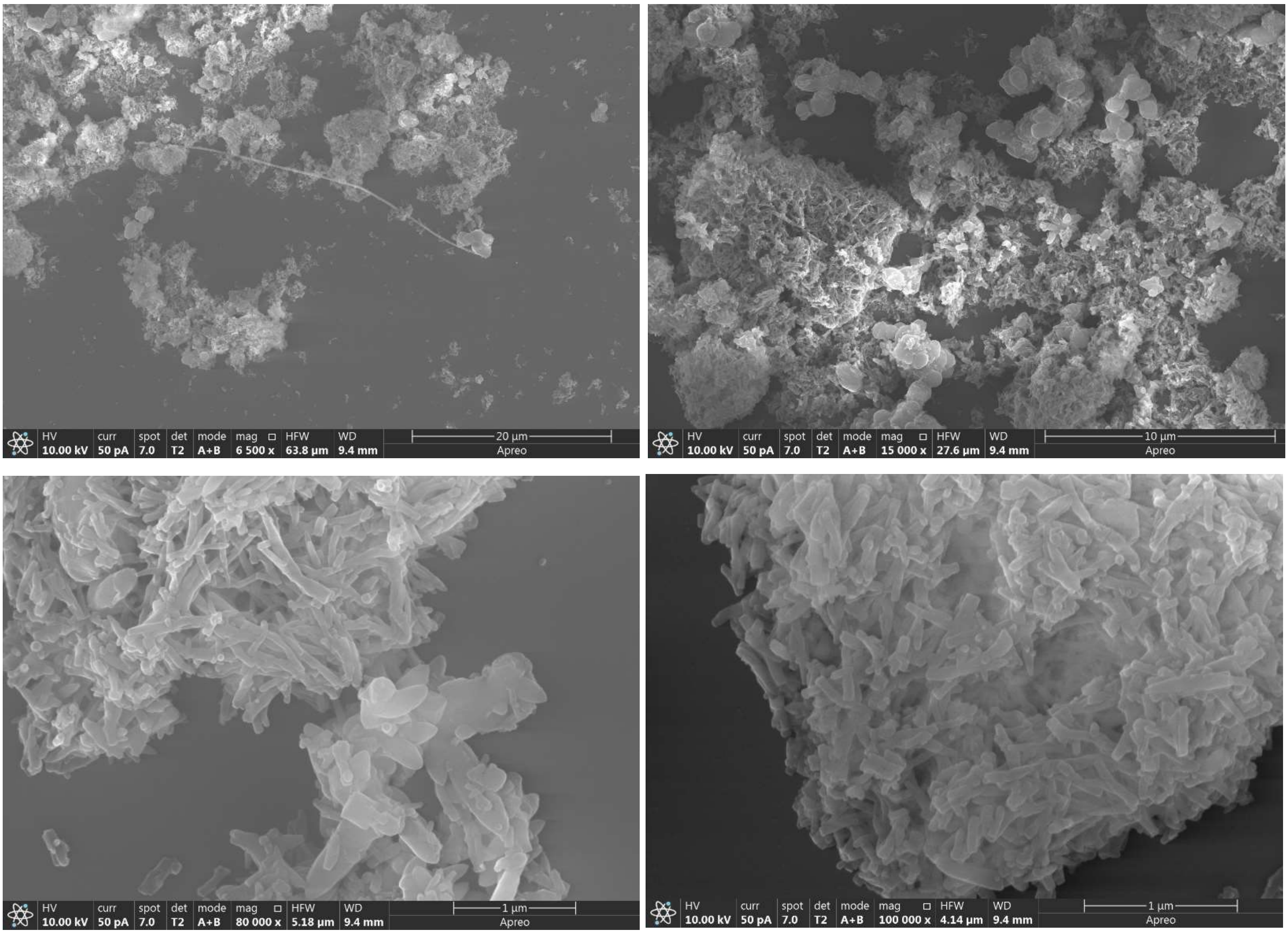

Figure S11. SEM images of the NH_4CN polymer synthetized at 170 ºC with a reaction time of 36 min.

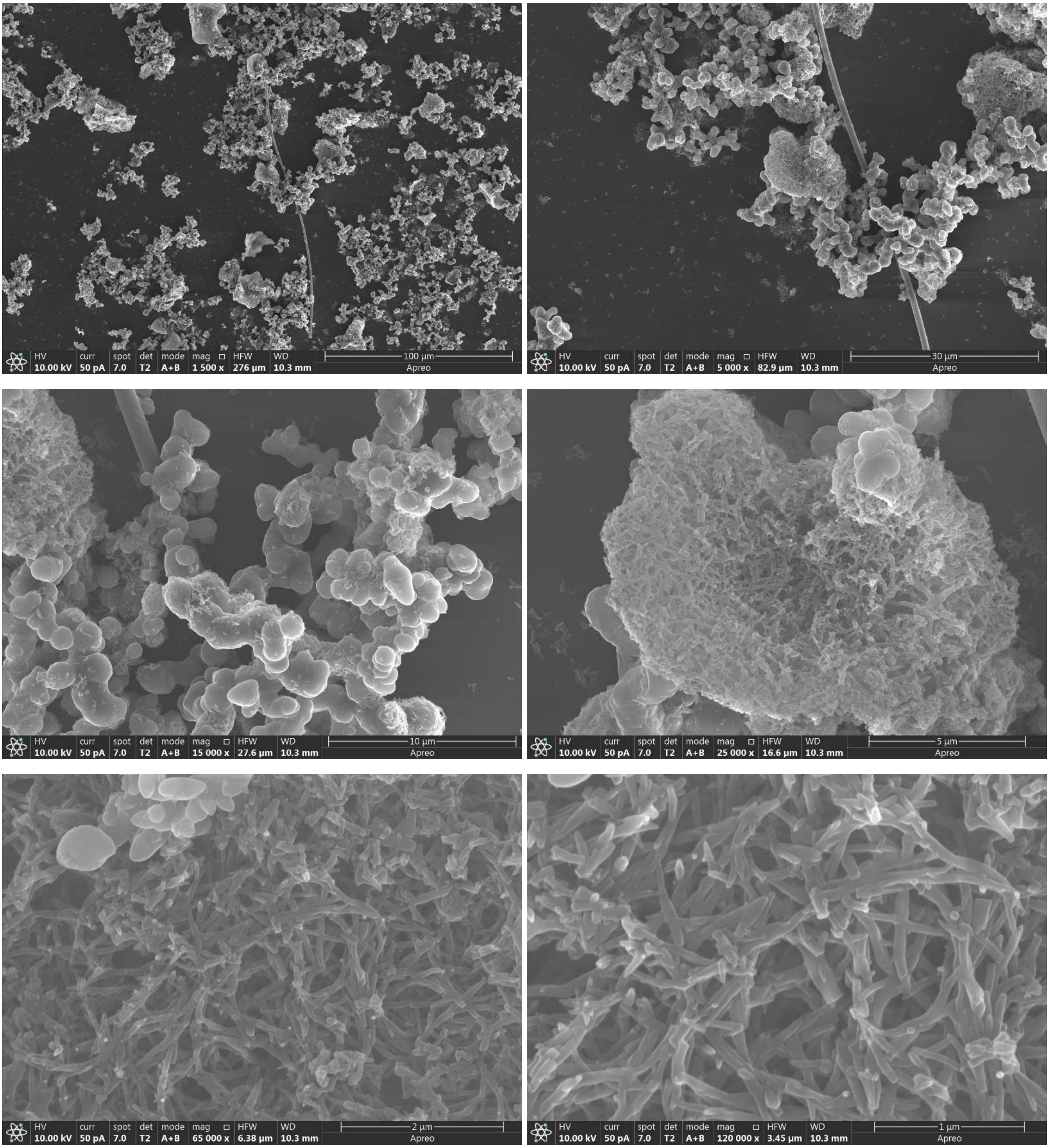

Figure S12. SEM images of the NH_4CN polymer synthetized at 170 ºC with a reaction time of 52 min.

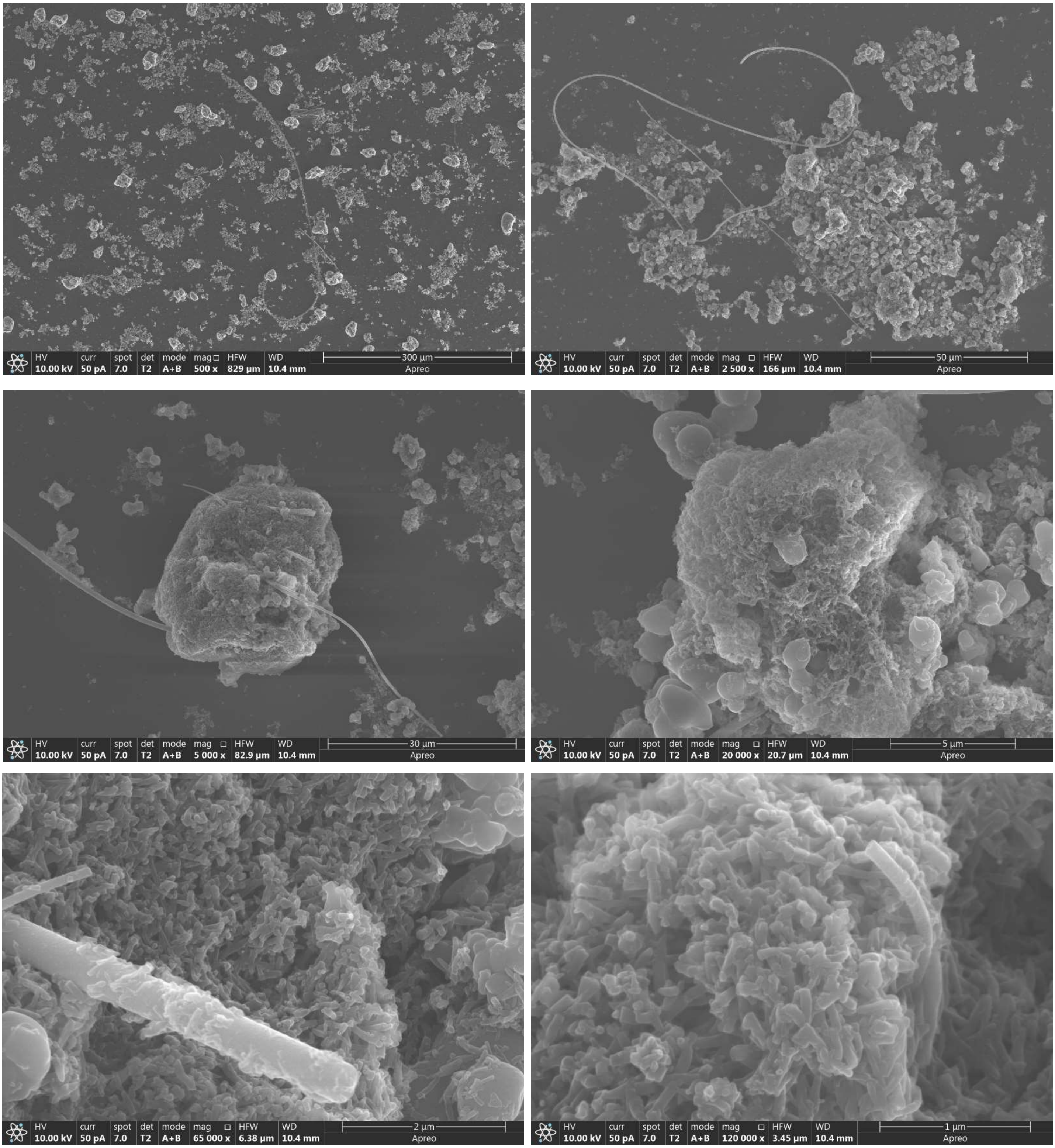

Figure S13. SEM images of the NH_4CN polymer synthetized at 170 ºC with a reaction time of 58 min.

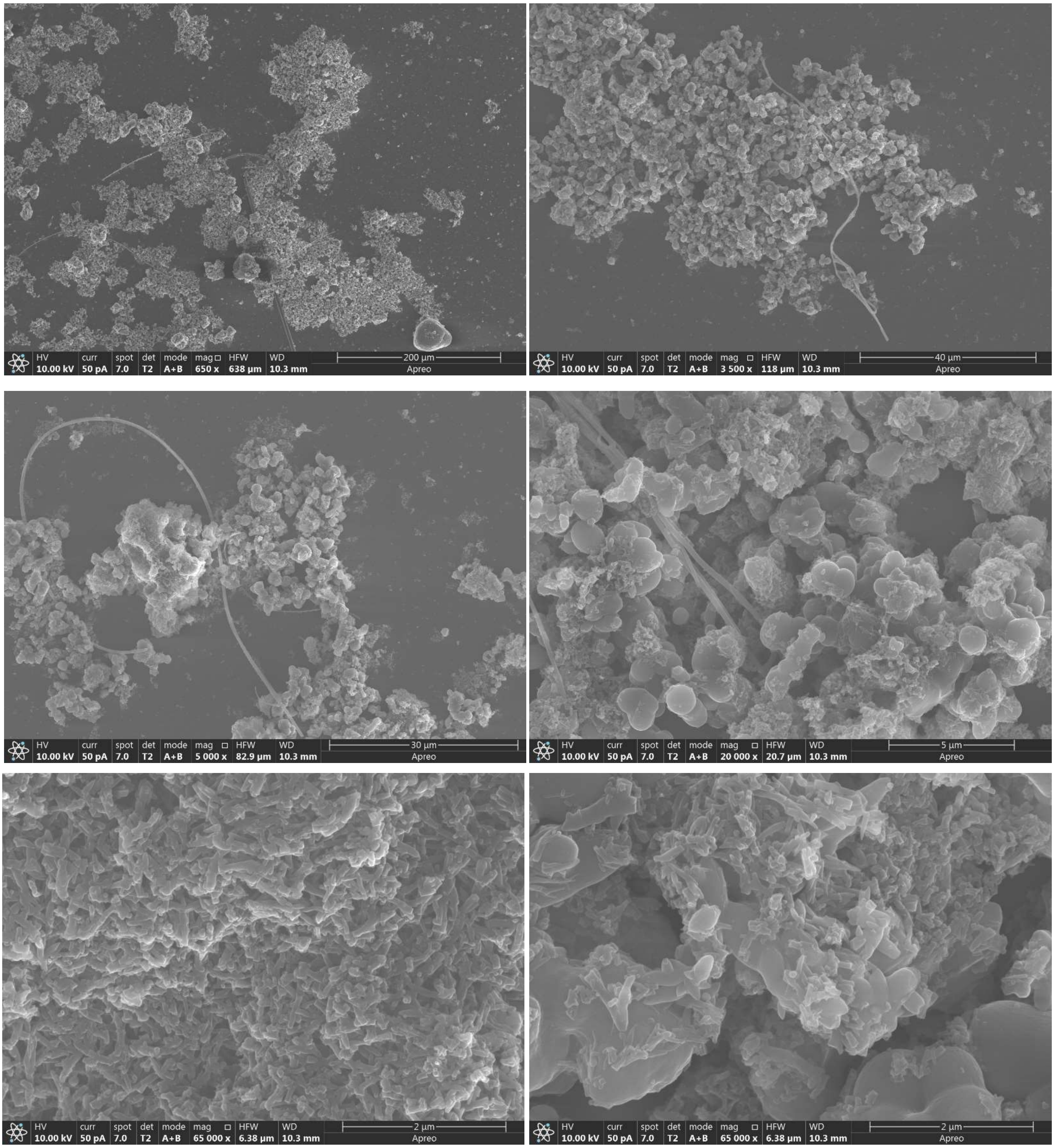

Figure S14. SEM images of the NH_4CN polymer synthetized at 170 ºC with a reaction time of 72 min.

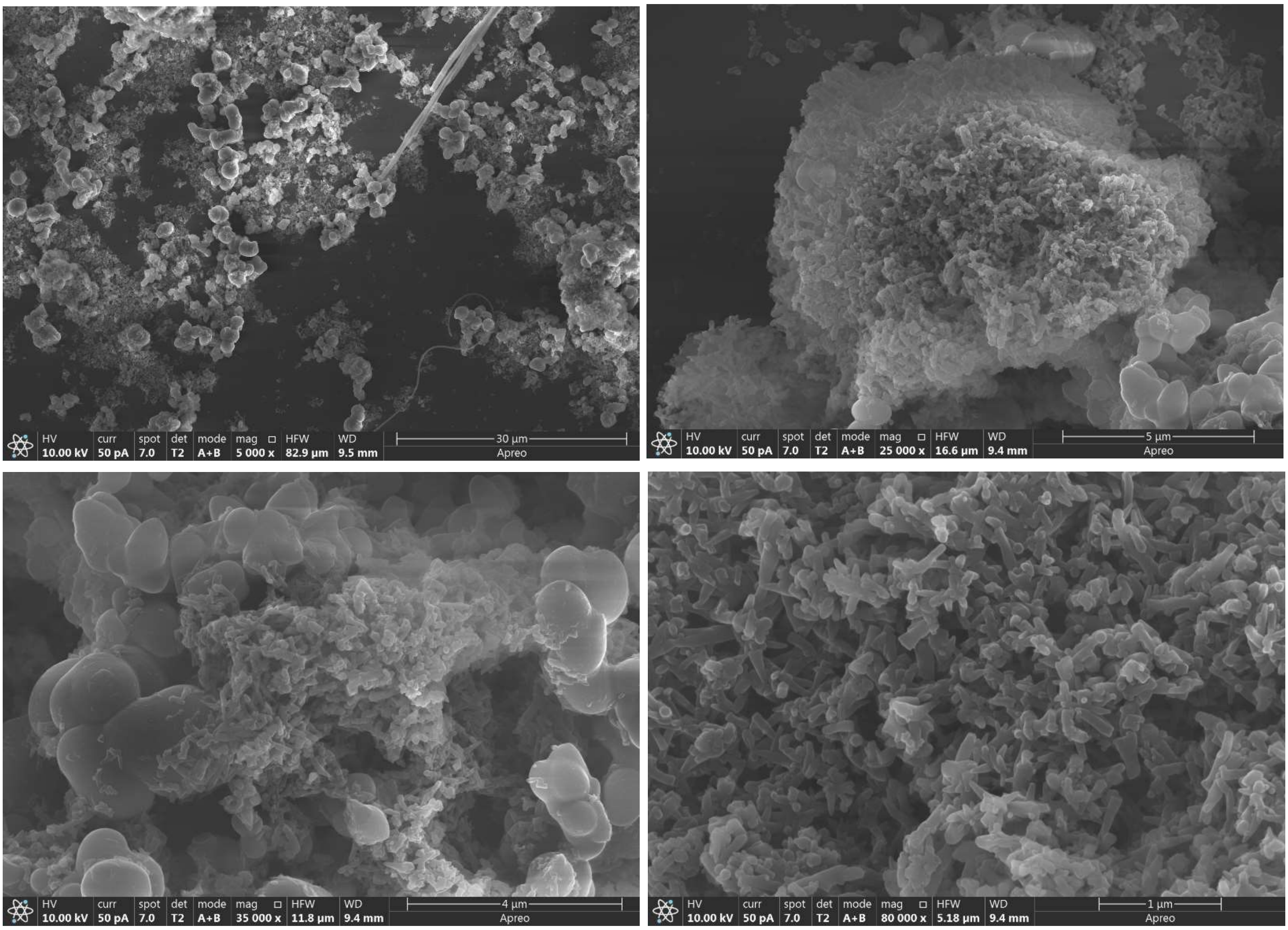

Figure S15. SEM images of the NH_4CN polymer synthetized at 170 ºC with a reaction time of 90 min.

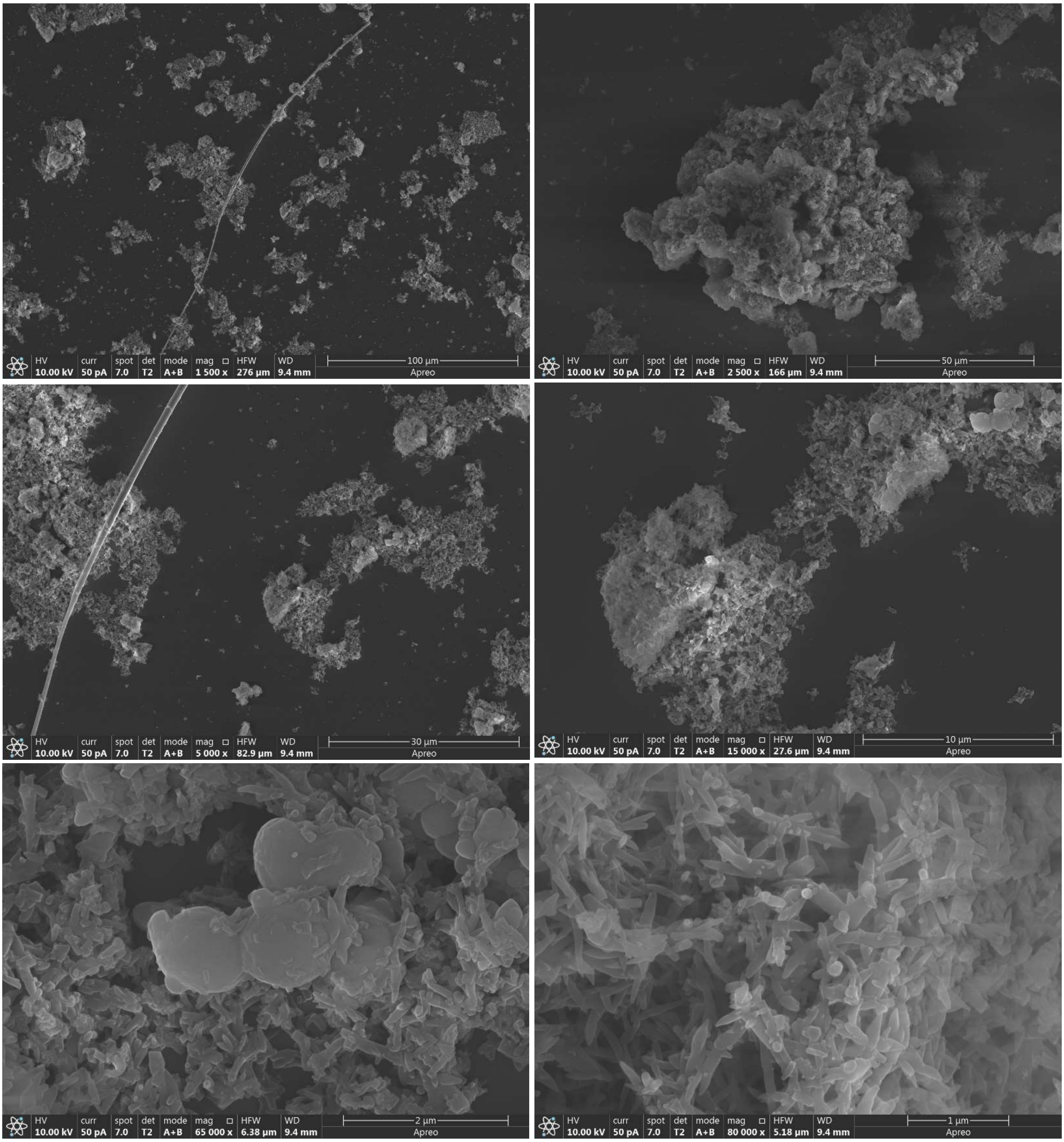

Figure S16. SEM images of the NH_4CN polymer synthetized at 170 ºC with a reaction time of 105 min.

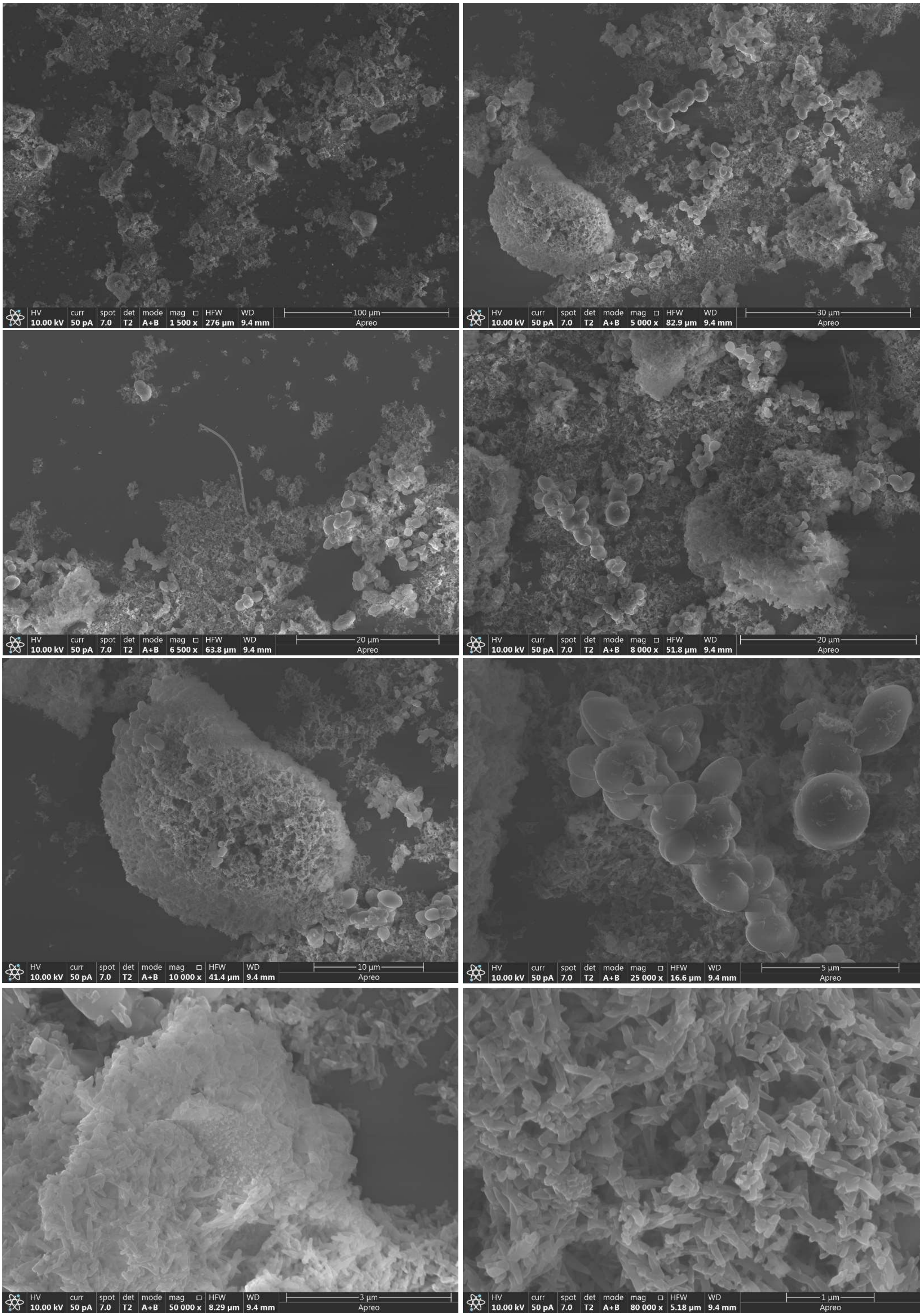

Figure S17. SEM images of the NH_4CN polymer synthetized at 170 ºC with a reaction time of 120 min.
